# A full-face mask for protection against respiratory infections

**DOI:** 10.1186/s12938-022-01027-1

**Published:** 2022-09-05

**Authors:** Chen-Hsun Weng, Chia-Lung Kao, Po-Wei Chiu, Shao-Peng Huang, Yuh-Shin Kuo, Yu-Yuan Lin, I-Chen Lin, Hung-Chieh Chang, Chien-Hsin Lu, Chih-Hao Lin

**Affiliations:** 1 Medical Device Innovation Center, National Cheng Kung University, No. 138, Shengli Rd., North District, Tainan, 70403 Taiwan; 2 Department of Emergency Medicine, National Cheng Kung University Hospital, College of Medicine, National Cheng Kung University, Tainan, Taiwan

**Keywords:** COVID-19, Mask, Airway, Infection protection

## Abstract

**Background:**

Aerosols and droplets are the transmission routes of many respiratory infectious diseases. The COVID-19 management guidance recommends against the use of nebulized inhalation therapy directly in the emergency room or in an ambulance to prevent possible viral transmission. The three-dimensional printing method was used to develop an aerosol inhalation treatment mask that can potentially prevent aerosol dispersion. We conducted this utility validation study to understand the practicability of this new nebulizer mask system.

**Results:**

The fit test confirmed that the filter can efficiently remove small particles. The different locations of the mask had an excellent fit with a high pressure making a proper face seal usability. The full-face mask appeared to optimize filtration with pressure and is an example of materials that perform well for improvised respiratory protection using this design. The filtering effect test confirmed that the contamination of designated locations could be protected when using the mask with filters. As in the clinical safety test, a total of 18 participants (10 [55.6%] females; aged 33.1 ± 0.6 years) were included in the final analysis. There were no significant changes in SPO_2_, EtCO_2_, HR, SBP, DBP, and RR at the beginning, 20th, 40th, or 60th minutes of the test (all *p* >.05). The discomfort of wearing a mask increased slightly after time but remained within the tolerable range. The vision clarity score did not significantly change during the test. The mask also passed the breathability test.

**Conclusion:**

The results of our study showed that this mask performed adequately in the fit test, the filtering test, and the clinical safety test. The application of a full-face mask with antiviral properties, together with the newly designed shape of a respirator that respects the natural curves of a human face, will facilitate the production of personal protective equipment with a highly efficient filtration system.

**Methods:**

We conducted three independent tests in this validation study: (1) a fit test to calculate the particle number concentration and its association with potential leakage; (2) a filtering effect test to verify the mask’s ability to contain aerosol spread; and (3) a clinical safety test to examine the clinical safety, comfortableness, and visual clarity of the mask.

## Background

Aerosols and droplets are the transmission routes of many respiratory infectious diseases, such as tuberculosis, influenza virus, rubella viruses (measles), varicella-zoster viruses (chicken pox), and coronavirus disease 2019 (COVID-19) [1–4]. Particles greater than 5 μm are removed from the air quickly and are thereby difficult to introduce into the respiratory tract [5]. Aerosol transmission is achieved by smaller respirable droplets and particles containing virus that can travel easily, be inhaled, and reach the lower respiratory tract [4, 5]. Aerosol-generating procedures (AGPs) include endotracheal intubation, cardiopulmonary resuscitation, tracheotomy, bronchoscopy, sputum suction, and inhalation therapy [6, 7]. Healthcare providers or other patients have a particular risk of infection when they are exposed to AGPs [6–8]. To reduce in-hospital transmission, various medical institutions are addressing concerns about AGPs and have taken various actions [9].

Personal protective equipment (PPE), which includes goggles, masks, gowns, clothing, gloves, and respirators, is an important tool to decrease infection spread [10–12]. Therefore, healthcare providers are required to wear PPE when performing AGPs. One of the major challenges they face in is the use of nebulizers for inhalation therapy. For patients with asthma or chronic obstructive pulmonary disease, it is important to use an inhalation bronchodilator to relieve symptoms during an acute attack. However, the COVID-19 management guidance recommends against the use of nebulized inhalation therapy directly in the emergency room or in an ambulance to prevent possible viral transmission [13]. Most guidelines recommend the use of alternative routes of administration, including breath-actuated nebulizers with exhalation filters or metred dose inhalers with valved holding chambers or in negative pressure areas [13–17]. Traditional nebulizer masks cannot prevent the spread of aerosols. Droplets of various sizes will be generated when patients receive inhalation therapy. Some innovative devices have been introduced for managing these AGPs [18–20], but these barriers still have limitations to their use with COVID-19 patients. Hence, we used a three-dimensional (3D) printing method to develop an aerosol inhalation treatment mask that can potentially prevent aerosol dispersion [16].

3D printing can be used as additive manufacturing introduces design and employment opportunities in hospitals [21]. 3D printing is behind the rise of point-of-care (POC) manufacturing [22, 23]. POC refers to the just-in-time creation of medical aids, such as surgical models, instruments, prosthetics, and other 3D printable items, to deliver better and lower-cost patient care [24]. Non-profit and for-profit hospitals, university engineering departments working with hospitals, government hospitals, and contract manufacturers with hospitals as clients are all adding POC additive manufacturing on site [25]. Even though additive manufacturing helps reduce hospital expenses and resources in the long term, administrators are still looking into ways to recoup the expense of additive manufacturing equipment and personnel more quickly. In addition, the health insurance industry is working on guidelines that support standards of care that will become reimbursable [25–27].

Surgical masks and N95 respirators are another piece of protective equipment that many companies and individuals are currently focusing on [13]. However, these systems need to receive regulatory approval before use, as many 3D printing items and/or materials may not provide the same level of barrier protection, fluid resistance, and infection control, especially when they are getting wet and encountering viruses [28]. Many research groups have found that the incorporation of drug molecules or copper oxide in the manufacture of multipurpose protective items (e.g. masks and goggles) provides additional antimicrobial and antiviral protection [29]. Patients with respiratory infections often generate large amounts of aerosols from coughing, sneezing, or heavy breathing [6]. Previous studies have shown an association between aerosol-generating procedures and healthcare providers who are infected with severe acute respiratory syndrome coronavirus 1 (SARS-CoV-1) [30]. Faced with the likely risk of airborne transmission and lack of proper protection, many healthcare professionals have developed their own equipment and guidelines to protect themselves from infection when working with patients [31].

**Fig. 1:**
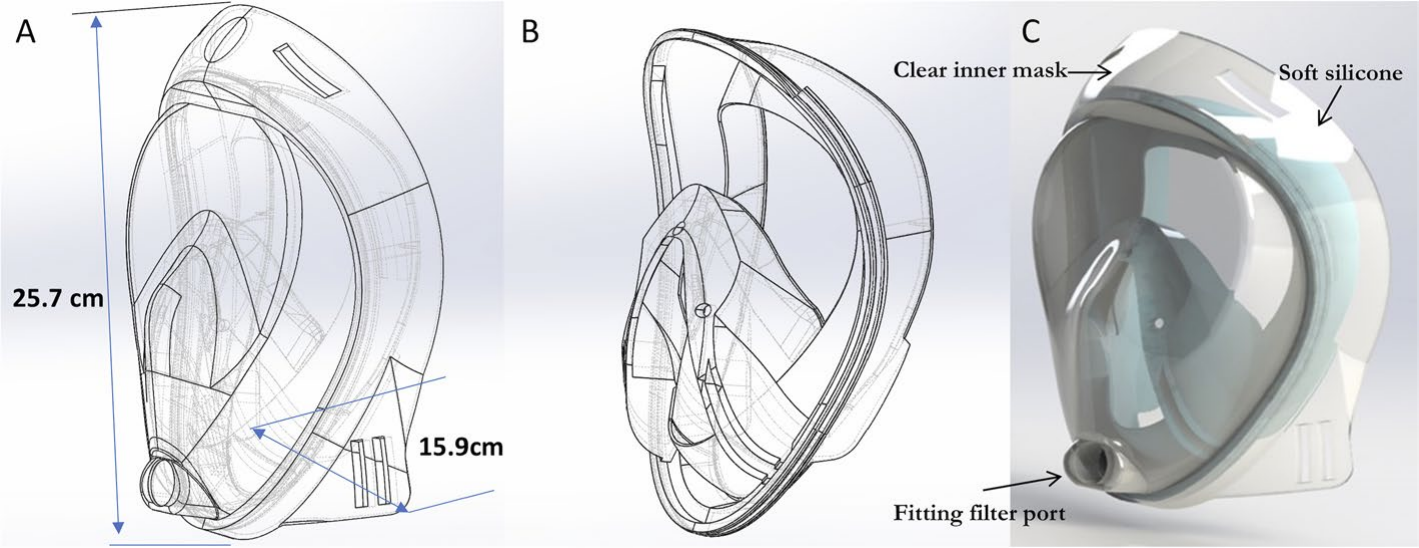
Schematic of the full-face mask for aerosol and droplet guarding and evaluation in protecting against respiratory infections. **A** The design of a clear inner mask with the length of 25.7 cm and the width of 15.9 cm. **B** The mould of soft silicone. **C** 3D rendering mould of the combination

Existing devices, such as the “intubations shield” are limited to a physical barrier of protection, as there are currently no strategies that provide protection from aerosol particles generated by a patient coughs and sneezes and by high-risk procedures performed by healthcare providers [32]. In the face of great urgency and severe risk, we designed, constructed, and tested the effectiveness of an affordable, scalable full-face mask for aerosol and droplet guarding and evaluated its ability to contain both droplets and aerosol particles (Fig. 1).

A Viper SLA-si2 (3D Systems, Rock Hill, SC, USA) 3D printer was used to fabricate a full-face mask in the polishing experiments [33]. The printing material used was WaterClear^®^ Ultra 10,122 from DSM Somos^®^, a resin with ABS-like properties. DSM’s Somos^®^ WaterClear Ultra 10,122 is a next-generation optically clear resin with good temperature resistance [34]. It produces colourless, functional, accurate parts that simulate an acrylic appearance. Somos^®^ WaterClear Ultra is a fast, low-viscosity, general-purpose resin [34]. The postprocessing of 3D-printed materials has been continuously studied, and with the recent expansion of the application of 3D printing, interest in it is increasing [35, 36]. Among various surface-machining processes, chemical mechanical polishing (CMP) is a technology that can effectively provide a fine surface via chemical reactions and mechanical material removal. The two-step polishing method can reduce the roughness, and we used sanding (#2000) and CMP sequentially (two-step polishing) to make the full-face mask clearer [36–38].

We designed and developed an innovative nebulizer mask [16]. This utility validation study was conducted to understand the fit quality, the filtering effect, and the clinical safety of this new nebulizer mask system.

## Results

### Test 1: The fit test

For each particle size distribution measurement, at least three runs were taken, and the average of the measured distributions was reported. The particle size distributions were measured by BT-610 (Met One Instruments, Inc., Oregon, USA). Studies of cough aerosols and of exhaled breath from patients with various respiratory infections have shown striking similarities in aerosol size distributions, with a predominance of pathogens in small particles (< 5 μm) [39]. We found that infectious aerosols are suspensions of pathogens in particles in the air, subject to both physical properties and airflow. Particle size is the most important determinant of aerosol behaviour. Particles that are 2 μm or smaller in size can remain airborne indefinitely under most indoor conditions unless there is removal due to air currents or dilution ventilation. We generated a positive reference material in the tent and stopped generating at 45 min. We measured the number of particles in the tent and the different sizes as shown in Fig. 4. We blocked the outlet of the full-face mask with a filter at 15, 30, and 45 min to observe the number of particles.

**Fig. 2:**
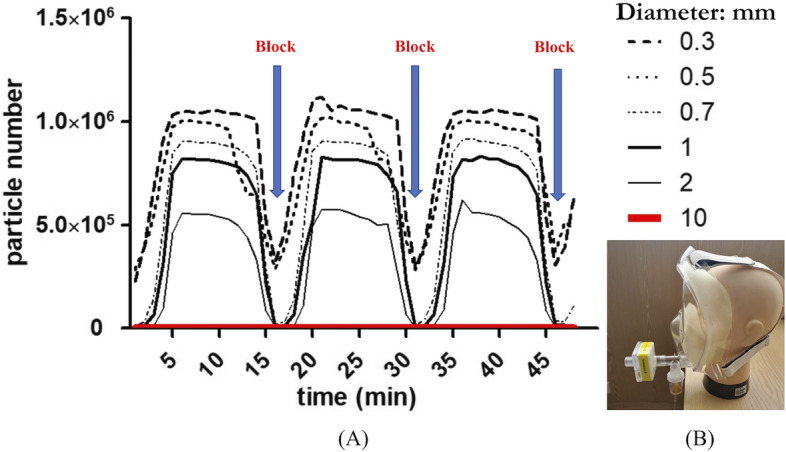
**A** The graph of particle numbers with time in the tent. **B** Using the filter to block the outlet

When we blocked the outlet with the filter, the number of particles was decreased in seconds as shown in Fig. 2. The full-face mask was experimented the protect wearers from breathing in hazardous contaminants in the air, especially the small particles like aerosol. This test confirmed that the filter can efficiently remove small particles.

**Fig. 3:**
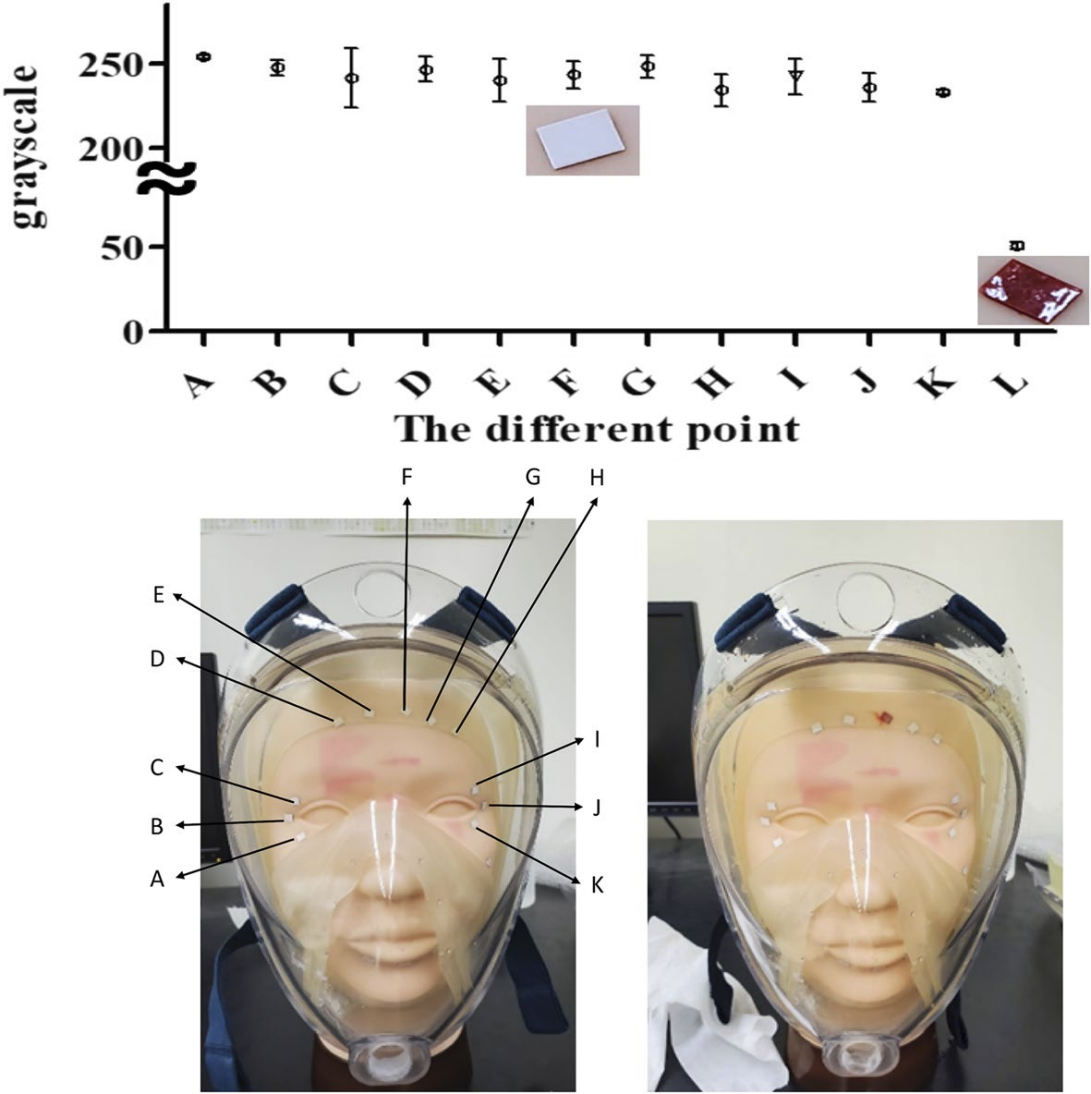
Photo of the stickers on the manikin head from A to K. The different points were analysed using ImageJ software to determine the grey of red. L is the photo of colour change with water

With the recognition that the leakage test is of particular importance to practise due to the high risk of infection, we tested an additional tool for nebulized inhalation therapy that helped consolidate learning and motivate change to practise in hospitals to use the full-face mask. We used a Damage Indicator Humidity Water Sensitive Colour Change Printing Adhesive Paper Sticker (Wida tech printing, Taiwan). We performed a simulated study to pressure test the mask with water to detect leakage, which is a common practice in industry. We put the full-face mask into the tank with water for 15 min. After 15 min, the colour of the paper sticker will change from white to red if the water infiltrates the mask. We put the stickers on different locations of the manikin head (from A to K), and the sticker L directly tested the water to check the colour change, as shown in Fig. 3.

**Fig. 4:**
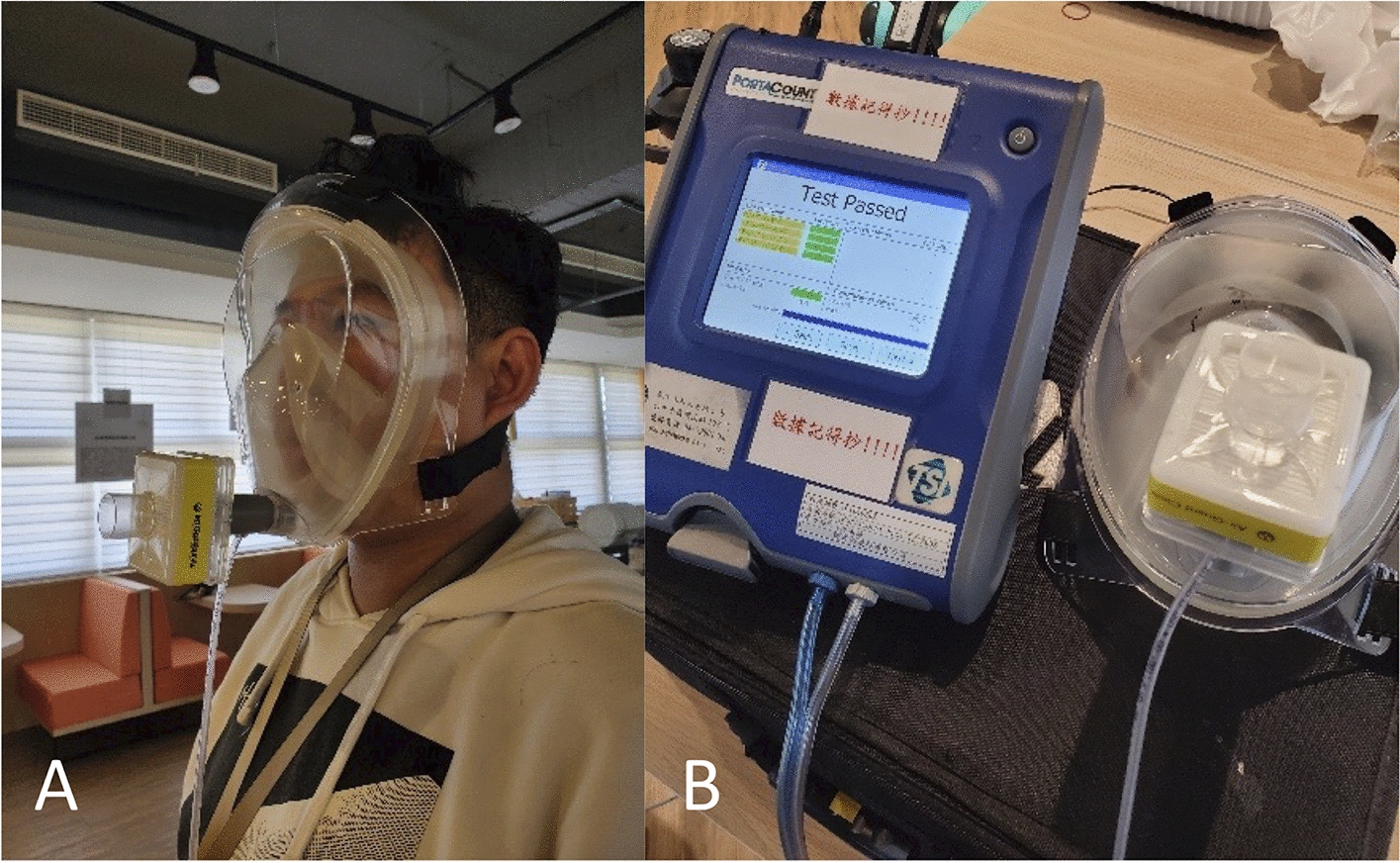
The breathability tests. **A** The participant underwent the four exercises using the fit tester. **B** The fit tester showed the test passed after the four exercises

**Fig. 5:**
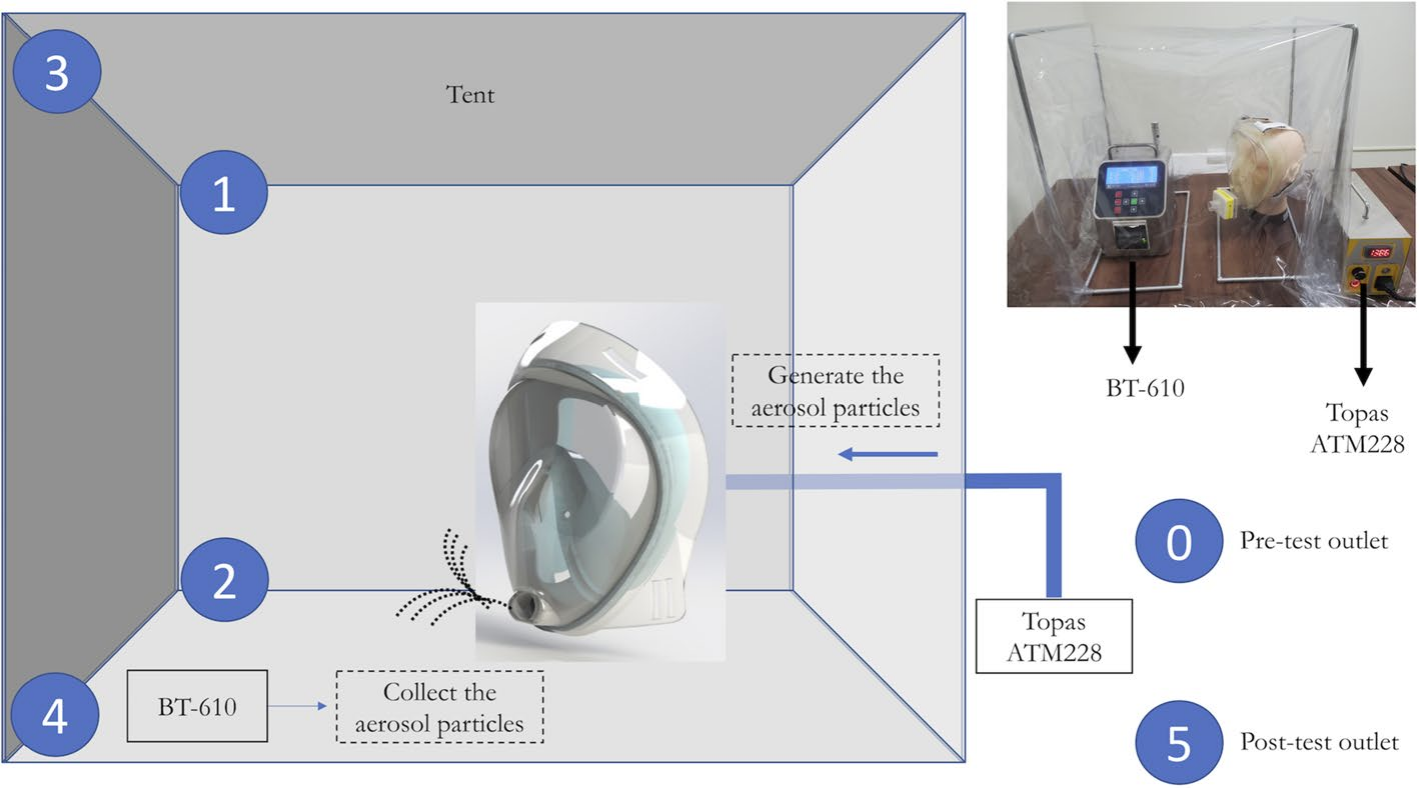
The experimental setup. Numbers 0–5 are the collect locations

The stickers from A to K did not have significant colour changes in the fit test. Figure 5 shows that the different locations of the mask had excellent fit with a high pressure making a proper face seal usability. The full-face mask appeared to optimize filtration with pressure and are examples of materials that perform well for improvised respiratory protection using this design.

### Test 2: The filtering effect test

The polymerase chain reaction (PCR) results showed the contamination of 5 locations: Nos. 0 and 5 were the samples from the outlet of the commercial aerosol generator, and Nos. 1, 2, 3, and 4 were set up on the tent. The overall contamination results of the tent are shown in Table 1. There were significantly more areas of contamination found on the tent without the filter than with.

**Table 1:** The polymerase chain reaction (PCR) results at different conditions (positive case/total test times)

Location No	With filter	Without filter
0	3/3	3/3
1	0/3	3/3
2	0/3	3/3
3	0/3	3/3
4	0/3	3/3
5	3/3	3/3

### Test 3: The clinical safety test

Twenty participants were included in this test, and no one dropped out of the study. However, 2 participants were excluded from the final analysis due to missing data. There were 18 participants, 10 females and 8 males, included in the safety test analysis. Their ages ranged from 24 to 49 years old, with a mean age of 33.1 ± 0.6 years old.

Table 2 shows the results of the safety test. There were no significant changes in oxyhaemoglobin saturation by pulse oximetry (SPO_2_), end-tidal carbon dioxide concentration (EtCO_2_), or heart rate (HR), systolic blood pressure (SBP), diastolic blood pressure (DBP), and respiratory rate (RR) at the beginning, 20th, 40th, or 60th minutes (all *p* > 0.05).

**Table 2:** The safety and comfort tests (*N* = 18)

Time	0 min median (IQR)	20 min median (IQR)	40 min median (IQR)	60 min median (IQR)	*p*
SPO2 (%)	98.0 (97.0–98.0)	98.0 (97.8–98.0)	98.0 (98.0–99.0)	98.0 (97.0–98.0)	0.29
EtCO2(mmHg)	33.0 (26.8–35.3)	28.0 (26.5–34.0)	30.0 (26.0–35.3)	30.0 (26.3–32.8)	0.49
SBP (mmHg)	125.5 (112.5–139.8)	115.5 (112.8–144.8)	127.5 (115.5–133.3)	129.0 (115.5–139.5)	0.90
DBP (mmHg)	80.0 (72.5–96.3)	76.5 (73.5–97.5)	78.5 (74.0–88.3)	81.0 (75.0–93.0)	0.72
HR (beats/min)	75.0 (70.0–80.5)	76.0 (70.3–81.3)	74.0 (69.0–81.0)	75.5 (67.0–79.75)	0.98
RR (beats/min)	19.0 (18.0–24.0)	20.0 (19.0–24.0)	21.0 (18.0–22.5)	19.0 (17.0–21.0)	0.05
Comfort score	2.0 (2.0–3.0)	3.0 (2.0–3.0)	3.0 (2.0–3.0)	3.0 (2.0–4.0)	0.02*
Vision clarity score	2.0 (1.8–2.3)	2.0 (2.0–3.0)	2.0 (2.0–3.0)	2.0 (2.0–3.0)	0.07

*IQR* interquartile range, *SPO*_*2*_ oxyhaemoglobin saturation by pulse oximetry, *EtCO*_*2*_ end-tidal carbon dioxide concentration, *SBP* systolic blood pressure; *DBP* diastolic blood pressure, *HR* heart rate, *RR* respiratory rate. Significant difference between the two groups

^*^p < 0.05, significantly different

The discomfort of wearing a mask increases slightly after time but remains within in the tolerable range. The comfort at 0 min and 60 min was significantly different. The most frequent complaint was facial discomfort, and no one complained of shortness of breath, dizziness, panic attacks, cold sweats, or chest tightness. The vision clarity score did not significantly change. The mask had no fogging, and clarity was not affected.

A total of 15 participants were enrolled in the breathability test using the bending-over exercise, the jogging-in-place exercise, the head side-to-side exercise, and the head up-and-down exercise. All the participants completed the testing process and passed the breathability test (Fig. 4).

## Discussion

The need for medical supplies has been a great challenge during the pandemic, and the lack of medical supplies has put healthcare providers and patients at risk. 3D printing technology can quickly supplement medical needs. We investigated whether full-face masks adapted with filters via the 3D printed method could provide the same protection as the currently recommended N95 with respect to particulate filtration and droplet deposition. Our study indicates that this new type of nebulizer mask is effective and safe. Potential benefits of such a filter are the universal availability and cost-effectiveness of reusable full-face masks.

This study also demonstrated that full-faced masks adapted as particulate filters can be used as a safe alternative to standard PPE combinations of N95 masks and eye protection. We used clear material to print and postprocess for emergency use. Fit testing confirms the fit of a full-face mask that forms a tight seal on the user’s face before it is used in the workplace. This ensures that users are receiving the expected level of protection by minimizing contaminant leakage into the facepiece. When a full-face mask does not fit properly, a portion of the air patients breathe can bypass the nebulizer mask with a filter and enter the patient’s airstream through breaks in the seal of the nebulizer mask. If this happens, healthcare workers may be exposed to harmful pathogens. In a similar vein, it is important to always wear the full-face mask with a nebulizer mask during exposure because even short periods of exposure substantially reduce the protection of healthcare workers.

We tested the safety and comfort of the mask with users. When wearing this mask, the user’s vital signs, including SPO2, EtCO2, HR, SBP, DBP, and RR, were stable. There was no statistically significant difference in these measures before and when wearing the mask. The comfort and clarity of vision were slightly affected but within an acceptable range. This result showed that wearing this mask does not increase the difficulty of breathing.

The nebulizer mask system that we designed can effectively filter the patient’s aerosols and droplets and is safe to use. This mask can be multifunctional. First, this new nebulizer mask, compared with the traditional one, can prevent the spread of aerosols. Second, this mask can provide an oxygen supply and thus can be used as a mobile isolation device when transferring patients with airborne diseases. Current practice is to use N95 masks during transfer; however, oxygen provision is a common clinical challenge when wearing N95 masks. Third, it can also be used as PPE. Different protection effects can be achieved by installing different filters on this mask, such as high-efficiency particulate air (HEPA) filters, activated carbon filters, and antidote filters. Moreover, 3D printing technology can be used for rapid production and can quickly respond to medical needs. Since the cost and timeliness of production were considered when designing this mask, we chose a size that is suitable for the average adult facial geometries. A single size mask cannot fit all face shapes, so customizing masks for different facial geometries or developing different sizes of masks would be the next step.

As the highly contagious Omicron coronavirus variant continues to spread, it is time to reconsider available face mask options. The Centres for Disease Control and Prevention is considering updating its mask guidance to recommend that people opt for the highly protective N95 or KN95 masks worn by healthcare personnel. With the highly transmissible Omicron variant driving record levels of infections and hospitalizations, masks of a higher quality than cloth coverings are recommended to protect against an airborne virus. This study examines how particles and aerosols move around the room depending on how people breathe and cough. Using simulations involving infected “SARS-CoV-2 reference material”, we measure the risks of exposure to demonstrate the safety and effectiveness of the full-face mask.

There is a risk of infectivity in COVID-19 recovered patients. With more confirmed COVID-19 patients recovering, a new challenge arises concerning the timing of emergency treatment for this patient group. A proportion of recovered patients may still be shedding virus. For emergent cases where nebulized inhalation therapy should proceed regardless, patients should be presumed to be infectious for at least 2 weeks after convalescence. The full-face mask could also play a role in risk stratification for these patients.

The related tests include the determination of the total inward leakage of a full-face mask, including the inward leakage of the air through the skin–facepiece interface. Based on the assumption that the total penetration of NaCl aerosol resulting from the mask leak is equivalent to the total penetration of the air, a measurement procedure was designed with the use of an indirect method of measurement. The designed measurement system was used to analyse changes in the pressure container, and the resulting data were subsequently used to identify the air leak through the skin–facepiece interface of the mask. During nebulized inhalation therapy, our innovative full-face mask can reduce the risk of COVID-19 transmission by ensuring that the full-face mask fits snugly against the patient’s face. Gaps can let air with respiratory droplets leak around the edges of the full-face mask. A full-face mask with a filter will stop more respiratory aerosols/droplets from getting inside the mask or escaping from the mask.

Our study has several limitations. First, real-world data on new mask use are still needed. Due to the limited resources, we did not perform comparison studies of this mask design with existing products on the market. This is crucial to assess its effectiveness over existing products. Second, we only tested the physiological parameters of wearing the mask for one hour, and longer wear requires more follow-up studies to confirm. Third, when used in inhalation therapy, the concentration of the drug and the real-world efficacy need to be further studied. Fourth, although we used biocompatible materials to make the mask, the current experiment did not test the compatibility between the material and the medications to be used. Finally, we only used the facial geometries of general adults for testing. Customization of masks to fit different facial geometries would be the goal of future development.

## Conclusions

The results of our study showed that this mask performed adequately in the fit test, the filtering test, and the clinical safety test. The application of a full-face mask with antiviral properties, together with the newly designed shape of a respirator that respects the natural curves of a human face, will facilitate the production of PPE with a highly efficient filtration system.

## Methods

We conducted three independent tests in this validation study: (1) a fit test; (2) a filtering test; and (3) a clinical safety test. This study was approved by the Institutional Review Board of National Cheng Kung University Hospital (B-ER-110-232).

### Test 1: The fit test

The aim of this test was to calculate the particle number concentration and its association with potential leakage. The fit of the mask was assessed by water pressure testing based on the visualization of stickers on a manikin head.

The schematic figure of the experimental setup is shown in Fig. 5. We used a commercial aerosol generator (model ATM228, Topas GmbH, Dresden, Germany) to generate positive reference material at 800 hPa. An atomizer aerosol generator (TOPAS ATM 228) was employed to generate sodium chloride (NaCl) aerosol particles from a 5 wt% solution prepared in Millipore water. NaCl droplets were dried by a silica dryer. The NIOSH NaCl method was used in this study to obtain respirator precertification [40, 41]. The valve leak NaCl and inhalation/exhalation tests were conducted to fulfil the NIOSH standards. The NaCl aerosol-based method is one of the most common testing methods for the face respirators meeting the requirements of the NIOSH protocol.

The particulate filtration efficiency method is led according to the American society of testing and materials (ASTM) F2299 protocol [42] and indicates the quality of the procedure/surgical masks. The particulate filtering efficiency follows Equation.

$$\mathrm{PFE}(\%)=\left(\frac{\mathrm{Cu}-\mathrm{Cd}}{\mathrm{Cu}}\right)\times 100,$$

where Cu and Cd are the averages of upstream and the downstream counts, the PFE results are between 1 and 99.99%. The higher the percentage represents better mask filtration. The particle size distributions were measured by BT-610 (Met One Instruments, Inc., Oregon, USA) which comply with 21 CFR Part 11 with a password feature to prevent unauthorized changes to the user settings. ISO 21501-4 calibration is available for ISO 14644-1 compliance. The ISO 21501-4 is the recognized standard for optical particle counter calibration. The ISO 14644-1:2015 specifies the classification of air cleanliness in terms of concentration of airborne particles in cleanrooms. The particle Counter meet ISO 14644-1:2015 criteria. It can also meet the calibration requirements of ISO 21501-4. Particle Counters can monitor this by documenting a count baseline of an area, detect when airborne particulate levels diverge from normal levels, and give an early warning of underlying issues, such as changes in operating procedures.

The Damage Indicator Humidity Water Sensitive Colour Change Printing Adhesive Paper Sticker (Wida tech printing, Taiwan) was used to ensure the safety, reliability, and leak prevention of the full-face mask. A leakage test is required for a water tank before use, and the full-face mask was placed into a water tank.

ImageJ software (US National Institutes of Health, Bethesda, Maryland, USA) was used for image mean grey value analysis [32, 43]. In the ImageJ software, the mean grey value within the selection is the sum of the grey values of all the pixels in the selection divided by the number of pixels. The grey layers are composed of pixels with values from 0 to 255. In the grey layers, a pixel value of 0 is pure black (dark). The higher the value is, the whiter (brighter) the layer. Therefore, on a grey layer, a pixel value of 255 is considered pure white. This mapping renders a two-dimensional matrix grey-scale photo image of the object. The fit of the mask was assessed by water pressure testing based on the visualization of stickers on a manikin head.

### Test 2: The filtering effect test

The aim of this test was to verify the mask’s ability to contain aerosol spread. The filtering effect test was using an aerosol generator to simulate the air particle dynamics and could illustrate the qualitative effectiveness and differences between two models with/without filter. To ensure a high quality of protection performance, laboratories must adhere to certain requirements comprising, for example, verification studies with commercially available tests, use of internal quality controls, and participation in external quality assessment (EQA) schemes. We used the available method for molecular-based SARS-CoV-2 testing [34]. The AccuPlex™ SARS-CoV-2 Reference Material Kit (SeraCare, Milford, MA, USA) was used as the test viral aerosol. In addition, the high similarity of positive reference material has the advantage of better virus detection and lower measurement uncertainty. The Reference Material Kit has been approved by the U.S. Food and Drug Administration (FDA) in conformance with local, state, and/or federal regulations or accreditation requirements and laboratory standard quality control procedures. The following external control materials are available: AccuPlex^™^ SARS-CoV-2 Reference Material Kit (Cat. No. 0505-0126). External positive controls were processed like patient samples to monitor RNA extraction, reverse transcription, PCR amplification, and detection.

First, two groups of 5 collectors (MF-Millipore^™^ Membrane Filter VSWP02500, Merck, Darmstadt, Germany) were set up with/without filters. One with no full-face mask and one with each of the full-face masks. Three intubation simulations were performed for each test. All the tests, the tent that was originally placed did not move. An easy, highly efficient, and sensitive method for monitoring airborne pathogenic microorganisms as well as viruses is the mixed cellulose ester (MCE) membrane method. The suitability of the water-soluble membrane, especially for virus sampling, has already been tested and proven. The membranes are particularly suitable because they show excellent virus collection efficiency with high retention rates. As soon as the membrane has been dissolved in deionized water or any other appropriate buffer or medium, all virus particles retained on the membrane can be further processed and detected using reverse transcription PCR (RT-qPCR).

To obtain fast, sensitive, highly specific, and reliable results on the presence of specific viruses, quantitative real-time PCR (qPCR) is the method of choice. Even sensitive RNA material can be detected by the combination of gelatine filtration and qPCR. However, RNA extraction should be considered a crucial step within sample preparation. Therefore, to reduce the loss of genetic material, attention should be given to this step. Several studies have shown that PCR analysis after air sampling using membranes provides recovery results superior to other detection methods and hence can be considered a precise and practicable method for the detection of airborne viruses.

The AccuPlex SARS-CoV-2 reference material kit (Cat. No. 0505-0126) was purchased from SeraCare. Primers and probes were synthesized by Tri-I Biotech (Taiwan). SuperScript^™^ III Platinum^™^ One-Step qRT-PCR Kit (Cat. No. 11732-020) was purchased from Thermo Fisher Scientific. Reliance One-Step Multiplex Supermix (Cat. No. 12010176) was purchased from Bio-Rad.

Viral extraction was performed using a Quick-RNA Viral Kit purchased from ZYMO RESEARCH. Two hundred microlitres of the sample (AccuPlex SARS-CoV-2) was added to the column and eluted in 15 μL DNase/RNase-Free water provided in the kit.

SuperScript^™^ III Platinum^™^ Taq Mix was used for the RT-qPCR with specific primers and probes tagged with FAM. The final concentration of primers in the reaction was 200 nM in a 50 μL reaction. The final concentration of the probe was 100 nM. Primer and probe sequences, as well as optimized concentrations are shown in Table 1. Thermal cycling was performed at 50 °C for 15 min for reverse transcription, followed by 95 °C for 2 min and then 40 cycles of 95 °C for 15 s, 60 °C for 30 s.

Reliance One-Step Multiplex Supermix was used for the RT-qPCR reaction with specific primers and probes tagged with FAM. The final concentration of the primers in the reaction was 200 nM in a 20 μL reaction. The final concentration of the probe was 100 nM.

The details of primers and probe sequences are provided in Table 3. The RT-qPCR setup is showed in Table 4.

**Table 3:** The primers and probe sequences

Primers	Sequence (5ʹ–3ʹ)	Annealing temperature
E_Sarbeco_F1	5ʹ-ACAGGTACGTTAATAGTTAATAGCGT-3ʹ	60 °C
E_Sarbeco_R2	5ʹ-ATATTGCAGCAGTACGCACACA-3ʹ	
E_Sarbeco_P1	5ʹ-ACACTAGCCATCCTTACTGCGCTTCG-3ʹ

**Table 4:** The RT-qPCR setup

Process	Temperature	Time	Cycles
RT	50 °C	15 min	1
Polymerase activation	95 °C	2 min	
PCR	95 °C	15 s	40
	60 °C	30 s	

**Fig. 6:**
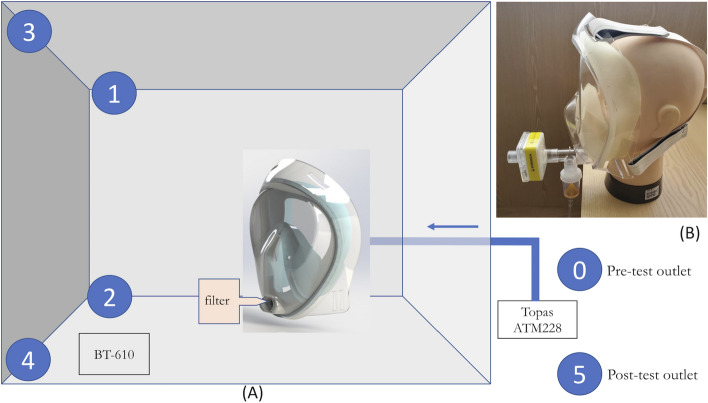
The validation of the filtering effect with different contamination locations. **A** Schematic of the experimental setup with a filter in the tent. **B** Photo of full-face mask when using nebulized inhalation therapy

The validation of the filtering effect with different contamination locations in this test is shown in Fig. 6.

### Test 3: The clinical safety test

The aim of this test was to examine the clinical safety of the mask. Individuals participated voluntarily, and informed consent was obtained from each of them. They were asked to wear the new nebulizer mask with a HEPA filter for 1 hour. The time of the test was during the daytime, and each participant had sufficient rest before the test. They did not perform intense activities during the test. The participants wore the masks while in a seated position. We monitored HR, BP, RR, SPO_2_, and EtCO_2_ at the beginning of the study and every 20 min after the test. BP and HR were measured on the participant’s left wrist with a wrist blood pressure monitor (Beurer BC 16, Beurer GmbH, Germany). SPO_2_ was tested with a fingertip pulse oximeter (OxiSmarter I, Acare Technology Co., Taiwan). EtCO_2_ and RR were checked by waveform capnography (EMMA^®^ Capnograph, Masimo Corp., USA). The blood pressure monitors, oximeters, and capnography sensors that were used in this study were calibrated regularly.

A self-rated, paper-based, 5-point (1 being the best and 5 being the worst) scoring evaluation questionnaire was obtained from the participants after the test to evaluate subjective comfort and vision clarity. The comfort score and vision clarity score were taken before the test and every 20 min after the test. The participants were free to quit the test at any time. If there was any discomfort during the test process, the participants were asked to record the reasons.

We also investigated the breathability of the device using the respiratory protection protocol [44, 45]. The test consisted of four exercises: (1) the bending-over exercise, with which the subject shall bend at the waist, as if going to touch his/her toes for 50 s and inhale 2 times at the bottom; (2) the jogging-in-place exercise, with which the subject shall jog in place comfortably for 30 s; (3) the head side-to-side exercise, with which the subject shall stand in place, slowly turning his/her head from side to side for 30 s and inhale 2 times at each extreme; and (4) the head up-and-down exercise, with which the subject shall stand in place, slowly moving his/her head up and down for 39 s and inhale 2 times at each extreme. The breathability test of the four exercises was evaluated using the PortaCount^®^ fit tester (model 8038, TSI Inc., Shoreview, MN, USA).

We used SPSS version 20 (IBM Corp. Released 2011. IBM SPSS Statistics for Windows, Version 20.0. Armonk, NY: IBM Corp) and a Wilcoxon signed-rank test to analyse the parameters at different times. The statistical significance was set at *p* < 0.05.

## Data Availability

All the data used in this paper are available in the figures and/or tables. The raw/processed data required to reproduce the findings of Tests 1 and 2 are available from the corresponding author upon reasonable request.

## Abbreviations

3D: Three-dimensional
AGPs: Aerosol-generating procedures
BP: Blood pressure
CMP: Chemical mechanical polishing
COVID-19: Coronavirus disease 2019
DBP: Diastolic blood pressure
EQA: External quality assessment schemes
EtCO_2_: End-tidal carbon dioxide concentration
FDA: Food and drug administration
HEPA: High-efficiency particulate air
HR: Heart rate
IQR: Interquartile range
MCE: Mixed cellulose ester
NaCl: Sodium chloride
PCR: Polymerase chain reaction
POC: Point-of-care
PPE: Personal protective equipment
qPCR: Quantitative real-time PCR
RR: Respiratory rate
RT-qPCR: Reverse transcription PCR
SARS-CoV-1: Severe acute respiratory syndrome coronavirus 1
SBP: Systolic blood pressure
SPO_2_: Oxyhaemoglobin saturation by pulse oximetry
